# Comment on: ‘Developing and paying for medicines for orphan indications in oncology: utilitarian regulation *vs* equitable care?’

**DOI:** 10.1038/bjc.2012.280

**Published:** 2012-06-28

**Authors:** S S Bielack

**Affiliations:** 1 Cooperative Osteosarkomstudiengruppe (COSS), Klinikum Stuttgart, Zentrum für Kinder- und Jugendmedizin – Olgahospital, Pädiatrie 5 (Onkologie, Hämatologie, Immunologie; Allgemeine Pädiatrie, Gastroenterologie, Rheumatologie) Bismarckstr. 8, Stuttgart D-70176, Germany

**Sir,**

In their minireview on developing and paying for orphan drugs in oncology, Davies *et al* (2012) used mifamurtide (MTP) to exemplify difficulties that could be encountered during the introduction of a new drug for a rare cancer. I was astonished to read that a suggestion, which was not mine, was erroneously linked to my name, and closely associated with what the authors consider perverse reasoning. Misquoting an editorial from the *European Journal of Cancer*, the authors write: ‘In this particular instance it could have been feared that the viability of the EURAMOS trial of established therapies would be impaired if more osteosarcoma patients were able to access MTP (Bielack, 2010). But this would be a perverse reason for delaying marketing approval for MTP, given the robust evidence of young adult survival advantage available’. (Davies *et al*, 2012).

Had such an argument been made, Davies *et al* (2012) would be quite close to the point. Suppressing the availability of a new active agent in order to not impede trials with old drugs would be decidedly unethical. However, the authors might want to reread the editorial and will then be able to confirm that it contains no statement even remotely associating access to MTP with success or failure of either the EURAMOS study or any other current osteosarcoma trial. One trial led to MTP licensing in Europe, but not in the United States. What Davies *et al* (2012) consider as ‘robust evidence of young adult survival advantage’ is considered by others as ‘not sufficient evidence of a survival advantage’ (US Food and Drug Administration, 2007), as evidence that does ‘not meet generally accepted standards for practice-changing conclusions’ (Hunsberger *et al*, 2008), or even as evidence that the drug is ‘ineffective and harmful’ (Prescrire International, 2011). Leading representatives of international osteosarcoma groups agree that additional clinical evaluations are required before the agent can be considered for routine use (Bielack *et al*, 2008). Had the authors included such thoughts, they might have concluded that the ‘to date partial introduction of the drug’, which they lament, much less ‘exemplifies barriers that the makers of orphan cancer medications have to overcome during the drug development and supply process’, but that it is rather the result of a considerable amount of scepticism regarding its efficacy. Of course, including such thoughts would have taken the discussion far away from utilitarian *vs* equitable to whether we should subject patients and health-care systems to the additional burdens associated with therapies that have not been evaluated nearly as thoroughly as one would like (Bielack, 2010).
